# Supplementary material for: The causal relationship between the human gut microbiota and pyogenic arthritis: a Mendelian randomization study
Source: Front Cell Infect Microbiol. 2024 Nov 26;14:1452480. doi: 10.3389/fcimb.2024.1452480 (PMC11629706; doi:10.3389/fcimb.2024.1452480)
Supplement: Supplementary file 1 [file DataSheet1.zip › mendelian test/ebi-a-GCST90027644.csv_leave_one_out.pdf]

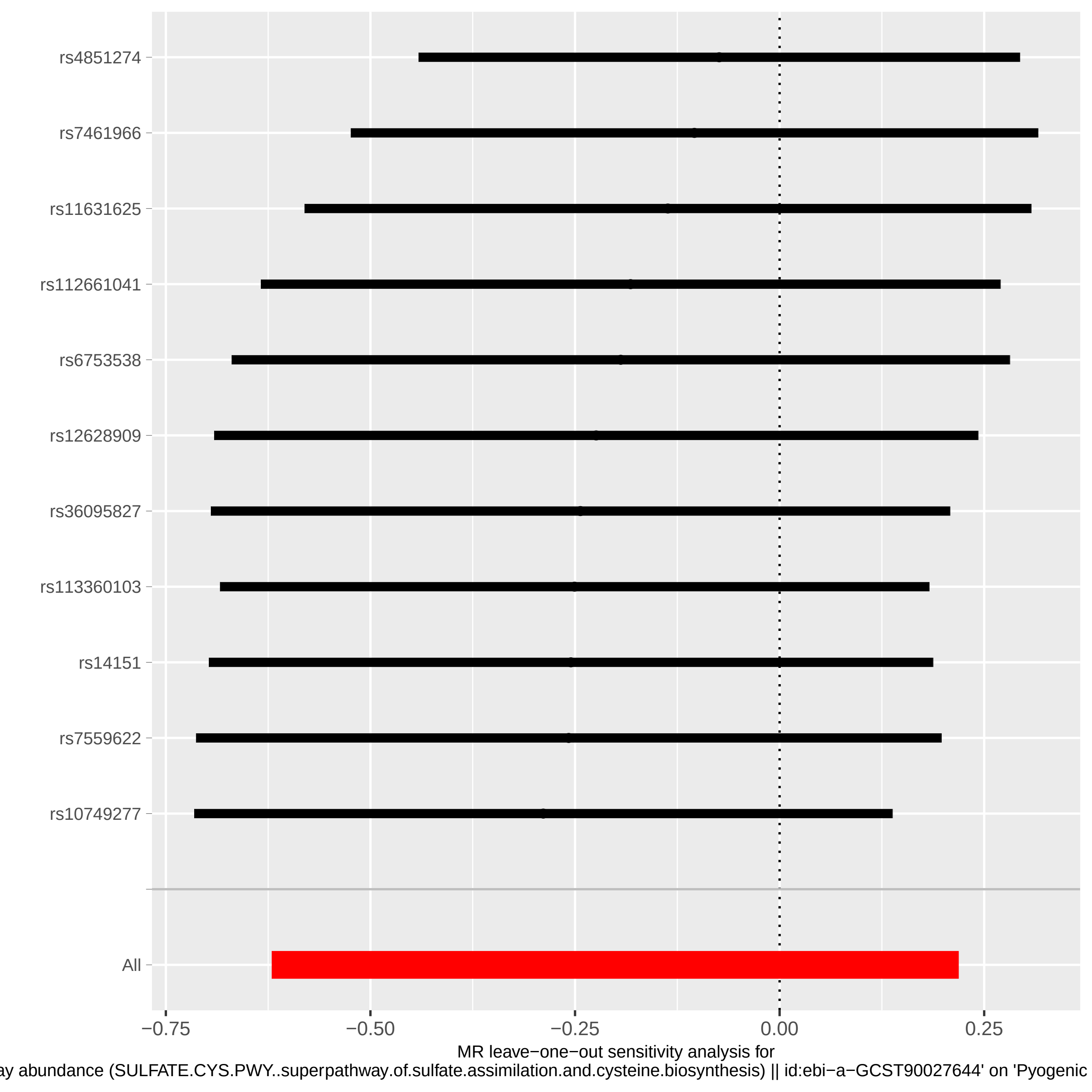

rs4851274
rs7461966
rs11631625
rs112661041
rs6753538
rs12628909
rs36095827
rs113360103
rs14151
rs7559622
rs10749277
All
−0.75
−0.50
−0.25
0.00
0.25
MR leave−one−out sensitivity analysis for
ay abundance (SULFATE.CYS.PWY..superpathway.of.sulfate.assimilation.and.cysteine.biosynthesis) || id:ebi−a−GCST90027644' on 'Pyogenic
